# Supplementary material for: High Frequency Haplotypes are Expected Events, not Historical Figures
Source: F1000Res. 2016 Feb 9;4:666. Originally published 2015 Sep 4. [Version 2] doi: 10.12688/f1000research.7023.2 (PMC4722684; doi:10.12688/f1000research.7023.2)
Supplement: Peer review history

# Peer review: Referee response for version 1

**DOI:** 10.5256/f1000research.7561.r11952

**Published:** 2016-01-18

**Authors:** Nick Patterson

**Affiliations:** 1 Medical and Population Genetics Program, Broad Institute of MIT and Harvard, Cambridge, MA, USA

This short note considers the recent paper by Balaresque *et al*. ^1^ on the distribution of Y-chromosome haplotypes in Central Asia. Through simulation they show that the haplotype frequency distribution is not very surprising and suggest that the results of ^1^ are most likely due to chance.

But there is more to the analysis of ^1^ than just the haplotype frequency. Their analysis groups haplotypes into `descent clusters', estimates the time to the most recent common ancestor (TMRCA) and looks into the spatial distribution of the haplotypes. None of this was simulated. There is no formal test applied in ^1^, but visually the results look to this reviewer very surprising under a scenario where to quote Guillot and Cox the results are

*chance proliferation of random male lines... from culturally undistinguished but biologically lucky...men*

In autosomal analysis of admixture events ^2^, overwhelming genetic evidence was found for the Mongol expansion across Eurasia. This by no means shows that the Y-chromosome signal was, at least partially, driven by high status Mongols, but to this reviewer this still seems more likely than not.

I have read this submission. I believe that I have an appropriate level of expertise to confirm that it is of an acceptable scientific standard, however I have significant reservations, as outlined above.

## References

[1] BalaresquePPouletNCussat-BlancSGerardPQuintana-MurciLHeyerEJoblingMA: Y-chromosome descent clusters and male differential reproductive success: young lineage expansions dominate Asian pastoral nomadic populations. *Eur J Hum Genet*.2015;23(10) : 10.1038/ejhg.2014.285 1413-22 10.1038/ejhg.2014.285 25585703PMC4430317

[2] HellenthalGBusbyGBBandGWilsonJFCapelliCFalushDMyersS: A genetic atlas of human admixture history. *Science*.2014;343(6172) : 10.1126/science.1243518 747-51 10.1126/science.1243518 24531965PMC4209567

# Peer review: Referee response for version 1

**DOI:** 10.5256/f1000research.7561.r11823

**Published:** 2016-01-15

**Authors:** Heather Norton

**Affiliations:** 1 Department of Anthropology, University of Cincinnati, Cincinnati, OH, USA

**Summary**

In this manuscript Guillot and Cox test the claim made by Balaresque *et al*. (2015) that a subset of Y-chromosome haplotypes from Central Asian men occur at “unusually” high frequency, possibly indicating social selection for men carrying these lineages. Using simulations designed to match the data reported by Balaresque *et al*. the authors demonstrate that the reported distribution of Y chromosome haplotypes can be obtained under neutral conditions. This suggests that it is not necessary to invoke a model that includes cultural transmission of reproductive success to explain the observed distribution.

**Comment**

While the focus of this correspondence article is on the Balaresque data, can the authors briefly comment on other papers that have also investigated cultural transmission of reproductive success—specifically, have there been other studies that report high frequency Y haplotypes in other populations that are **not** consistent with neutrality?

I have read this submission. I believe that I have an appropriate level of expertise to confirm that it is of an acceptable scientific standard.

# Peer review: Referee response for version 1

**DOI:** 10.5256/f1000research.7561.r10223

**Published:** 2015-10-01

**Authors:** Sohini Ramachandran

**Affiliations:** 1 Department of Ecology and Evolutionary Biology, Brown University, Providence, RI, USA

Guillot and Cox present a very interesting criticism of Balaresque *et al*.'s work in press on high frequency haplotypes in Central Asian Y chromosomes, by showing that distributions like those observed by Balaresque and colleagues can be observed using neutral simulations.

I have three comments I would like to see the authors address:

1. The "Genghis Khan reproductive success" hypothesis emerged in Zerjal *et al*.'s work in 2003 and I think it would be helpful for the authors to comment on what analyses in that work support Zerjal *et al*.'s conclusions; can their simulations reproduce what Zerjal *et al*. observed under strictly neutral processes without a high number of mergers in the coalescent process?
2. The phrase "historically unrecorded, culturally undistinguished, but biologically lucky Central Asian men." should be changed to "historically unrecorded but biologically lucky Central Asian men."
3. The authors should provide sample output files for their simulation pipeline for users to analyze, and their code so that the number of simulations run is a user-provided argument. Given that the pipeline can take at least hours and perhaps days to generate the million simulations they studied, a toy example is worth looking at quickly and the authors could allow readers to generate examples more quickly without needing to fiddle with the bash/python/R pipeline on their own.

I have read this submission. I believe that I have an appropriate level of expertise to confirm that it is of an acceptable scientific standard, however I have significant reservations, as outlined above.
